# Cultivar and origin authentication of ‘Fuji’ and ‘gala’ apples from two dominant origins of China based on quality attributes

**DOI:** 10.1016/j.fochx.2024.101643

**Published:** 2024-07-14

**Authors:** Lixue Kuang, Zhiqiang Wang, Yang Cheng, Haifei Li, Jing Li, Youming Shen, Jianyi Zhang, Guofeng Xu

**Affiliations:** Research Institute of Pomology Chinese Academy of Agricultural Sciences, Xingcheng, Liaoning Province 125100, PR China

**Keywords:** Apple, Quality attribute, Cultivar authentication, Origin authentication, MLP–NN

## Abstract

Apple quality is closely related to its cultivar and origin. However, the apple quality characteristics of different cultivars and origins are unclear. The hypothesis that some quality indicators can effectively distinguish the cultivar and origin of apples. The result indicated that the discriminant accuracy of the models was above 90%, and the multilayer perceptron neural network (MLP–NN) model was superior to the linear discriminant analysis (LDA) model. The identification accuracy of cultivars was higher than origins. The main reason was that the single fruit weight, vitamin C, total soluble solid, soluble sugar, sweetness value, sorbitol, glucose, fructose, sucrose, malic acid, quinic acid and citric acid of ‘Fuji’ apples were significantly higher than ‘Gala’ apples. This study provides a foundation for the quality evaluation and further geographical traceability studies of apples. Further studies related to the regulatory mechanism of environmental conditions on apple quality characteristics should be explored for theoretical confirmation.

## Highlights

• Quality attributes of ‘Fuji’ and ‘Gala’ apples from two dominant origins in China were compared.
• Different discriminate models were constructed for apple cultivar and origin authentication.
• The MLP–NN model obtained higher training and validation accuracies than LDA model.

## 1 Introduction

The apple (*Malus × domestica Borkh*), as a member of the Rosaceae family, is one of the most important commercial fruit crops grown in temperate regions around the world (Xu et al., 2020). In 2022, Chinese apple production was 47.573 million tons, contributed 49.37% of worldwide apple production (FAO, 2024). The Loss Plateau (LP) and Bohai Bay (BB) regions are the two major apple origins in China, accounting for 52% and 33% of China's total apple production and 58% and 24% of China's total apple planting area, respectively (Zhang et al., 2019; Zhang, 2022; Zhang, Shen, Ma and Xu, 2023). Currently in China, ‘Fuji’ apple is the most dominant cultivar, accounting for 72.7% of total apple production (Chen et al., 2020). Although the production of ‘Gala’ is <10% of total apple production, it is the representative of main early–maturing apple cultivar.

The supply and consumption of quality fruit is an important indicator of a country's social development and standard of living (Yang et al., 2023). The apple is one of the most popular fruits in our life. The quality of apples mainly includes sensory, flavor, and nutritional qualities. Sensory quality mainly includes external characteristics such as fruit size, shape, color, texture, aroma, and surface defects; Flavor quality mainly includes characteristics that affect the taste, such as soluble solid content, titratable acid content, solids–to–acid ratio, various sugar components and organic acids; Nutritional quality mainly includes nutrients such as vitamin, phenol and mineral matter. With the continuous improvement of living standards and consumption capacity, more and more consumers like to pay a high price to buy those beautiful, delicious and nutritional apples.

The quality of apples is closely related to the cultivars and origins. Apples of different cultivars and geographical regions are believed to have different internal and external qualities and the apples from quality cultivars and areas often demand higher prices. However, some dishonest traders often mix and replace these special local products with cheaper or inferior ones to deceive high profits. Therefore, clarifying the apple cultivar and origin characteristics is essential for ensuring quality and protecting consumer interests, and has attracted the attention of more and more researchers (Kuang et al., 2020).

The quality of ‘Fuji’ apples was widely studied due to its dominant position in the apple market of China (Kuang et al., 2020; Zhang, 2022). However, the evaluation for quality properties and physiochemical characteristics of ‘Gala’ apples is lacking and the quality differences between ‘Fuji’ and ‘Gala’ apples grown in different cultivation regions are not fully clear. In addition, the apples from different cultivars or origins can be easily mixed during harvesting and marketing. Therefore, effective and reliable technologies that could identify apple cultivar and geographical origin are demanded urgently by sellers and consumers. To fulfill these gaps, the quality parameters differences between ‘Fuji’ and ‘Gala’ apples grown in different cultivation regions were investigated and compared in this study. Linear and nonlinear discriminate models were constructed for apple cultivars and origins simultaneous authentication. The discrimination accuracy of different models was compared. This study provides a foundation for the quality evaluation and further geographical traceability studies of apples.

## 2 Materials and methods

### 2.1 Sample collection

The BB region included Shandong, Hebei and Liaoning Provinces. The LP region included Shaanxi, Shanxi, Gansu, Ningxia, Henan and Xinjiang Provinces. A total of 177 ‘Fuji’ apple samples were collected at the harvest maturity stage, including 85 samples from BB region (Hebei, *n* = 38; Shandong, *n* = 36; Liaoning, *n* = 11) and 92 samples from LP region (Shaanxi, *n* = 34; Shanxi, *n* = 19; Gansu, *n* = 10; Ningxia, *n* = 8; Henan, *n* = 6 and Xinjiang, *n* = 15). A total of 77 ‘Gala’ apple samples were collected at the harvest maturity stage, including 26 samples from BB region (Hebei, n = 6; Shandong, *n* = 13; Liaoning, *n* = 7) and 51 samples from LP region (Shaanxi, *n* = 26; Shanxi, *n* = 25). For each sample, about 20 kg of apple fruits at harvest maturity were collected from at least 10 trees and transported to the laboratory immediately.

### 2.2 Sample preparation

First, ten apples were selected randomly from each sample for the determination of single fruit weight, fruit type index and flesh firmness. About 3.0 kg of fruit was ground into a powder in liquid nitrogen by a freeze grinder (SPEX 6875D, CEM, USA) and stored at −20 °C for the determination of sugar components, organic acids, total phenolic and total flavonoid content. The rest samples were homogenized by a homogenizer (JT–C, Tianjin, China), which was used for the determination of total soluble solid (TSS), soluble sugar (SS), titratable acidity (TA) and vitamin C (Vc) content.

### 2.3 Physicochemical analyses

In order to determine the average single fruit weight, the apples were weighted one by one using an electronic balance (PL602–L, Mettler Toledo, Switzerland). The fruit type index is the ratio of vertical and horizontal diameter, which was measured by a vernier caliper (0–300 mm, Mitutoyo, Japan). After removing the peel, the flesh firmness of apple sample was measured on two sites from both sides of equator using a penetrometer (GY–4, HANDPI, China).

The TSS content was measured by a Digital display sugar refractometer (PAL–1, ATATO, Japan) with three replicates. The content of TA was determined by the Automatic potentiometric titrator (904 Titrino, Metrohm, Switzerland) according to the method of Kuang et al. (2020). The determination of SS and Vc was conducted with the 3, 5–dinitrosalicylic acid colorimetry method and 2, 6–dichlor–oindophenol (2, 6–D) solution titration method, respectively (Ministry of Agriculture, People's Republic of China, 2015; National Health Commission of the People's Republic of China, 2016). Detailed procedures for the determination of TA, SS and Vc were described in the supplementary materials.

The extraction and determination of soluble sugar components, organic acids, total phenolic and total flavonoid content were carried out according to the previously reported method (Shafi et al., 2019; Zhang, 2022). Detailed procedures for sample extraction and determination were provided in the supplementary material. The soluble sugar components (including sorbitol, glucose, fructose, and sucrose) were detected by an Ion chromatograph (ICS–5000, Dionex, USA) with a conductivity detector, an anion exchange analytical column (Dionex CarboPacTM PA10, 4 mm × 250 mm, Thermo Fisher Scientific, USA), and a guard column (IonPac AG23, 4 mm × 50 mm, Dionex, USA). Four organic acid compounds, including quinic acid, malic acid, Citric acid and shikimic acid were detected by HPLC (LC–10 A, Shimadzu, Japan), with SPD–10 A UV–VIS detector, and a C18 column (Ultimate Loss Plateau–C18, 4.6 mm × 300 mm, 5 μm, Ultimate, China).

The total phenolic and flavonoid content were determined by the Ultraviolet visible spectrophotometer (U3900, Hitachi, Japan). Absorbance of total phenolic was measured at 765 nm using a spectrophotometer. The results were expressed as milligrams GA equivalent (GAE) per kilogram of fresh weight (FW). The absorbance of total flavonoid was measured at 500 nm, results were expressed in terms of the catechin equivalent CE (mg/kg).

According to previous researches (Mignard et al., 2022; Zhang, 2022; Zheng et al., 2015), the SS in apples mainly included sorbitol, glucose, fructose, and sucrose. So we chose the 4 sugars to calculate the sweetness value (SV). The SV of each sample was calculated according to eq. (1):

(1) $$\mathrm{SV}=C_{\text{sucrose}}\times 1+C_{\text{fructose}}\times 1.75+C_{\text{glucose}}\times 0.7+C_{\text{sorbitol}}\times 0.4$$

where, SV was the sweetness value. C_suc_, C_fru_, C_glu_ and C_sor_ was the content of sucrose, fructose, glucose and sorbitol in samples. The sweetness intensity of sucrose, fructose, glucose and sorbitol was 1, 1.75, 0.7 and 0.4, respectively (Li et al., 2021). In addition, the ratios of total soluble solid to titratable acidity (RTT), soluble sugar to titratable acidity (RST) and sweetness value to titratable acidity (SVT) were calculated to evaluate the quality of apples.

### 2.4 Statistical analysis

The obtained data was analyzed using SPSS 20.0 package. One–way ANOVA at the 5% significance level and Duncan's multiple comparisons was performed to determine the significant differences for the mean concentrations of each indicator in the different fruit samples. Principal component analysis (PCA) was used for dimensionality reduction. LDA and MLP–NN were performed to establish discriminant models for identifying the sample species. LDA is a dimensionality reduction technique for supervised learning, which means that each sample of its dataset has a class output. This is different from PCA. PCA is an unsupervised dimensionality reduction technique that does not consider the output of sample categories. The LDA model was established by stepped–linear discriminant analysis and the cross–validation was adopted to verify the accuracy of the models. The MLP–NN model for information processing using a structure similar to the synaptic connections of the brain, and it has nonlinear dynamic properties. The MLP consists of formal neurons and connections between them. The neurons are arranged in layers (an input layer, one or more hidden layers, and an output layer), and the connections are unidirectional from the input to the output. Adjacent layers are fully connected, and no connections exist between neurons within the same layer. In the study, the samples were divided into a training data set (184 samples) and a test data set (70 samples). We used an MLP with three layers according to the architecture 21 × 3 × 4: an input layer with 21 neurons, a hidden layer with 3 neurons, and an output layer with 4 neurons, corresponding to the classes to be assigned (Gala–LP, Gala–BB, Fuji–LP, and Fuji–BB). LDA and MLP–NN were performed by the SPSS 20.0 package. PCA was performed and the graphics were drawn using Origin 2021 Version.

## 3 Results

### 3.1 Sensory characteristics of different apple cultivars and origins

**Fig. 1:**
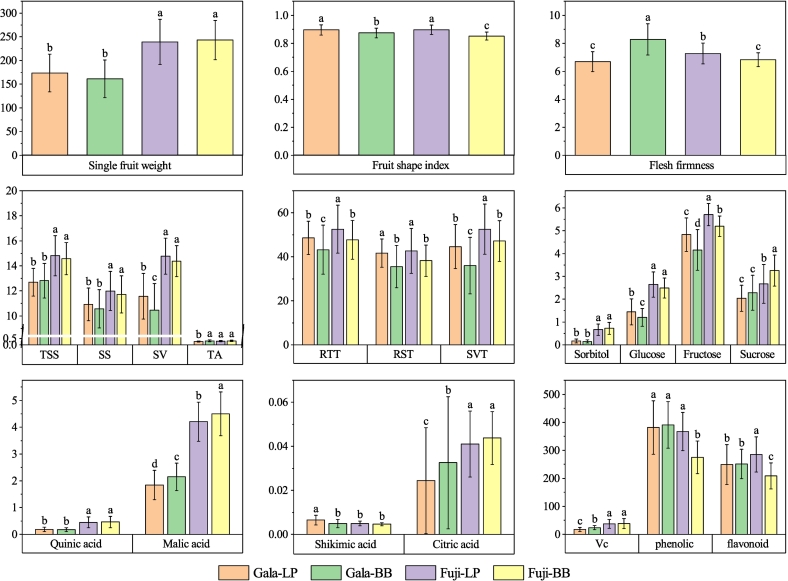
The ANOVA result of ‘Fuji’ and ‘Gala’ apples from Loss Plateau and Bohai Bay regions.

Quality grading of apples is an important item in post–harvest handling and marketing, it is mainly performed according to sensory characteristics of apples. In the sensory quality of apples, fruit size is a very important evaluation index and mainly described by weight. Table S1 and Fig. 1 show the quality of ‘Fuji’ and ‘Gala’ apples from Loss Plateau (LP) and Bohai Bay (BB) regions. There was significant difference regarding single fruit weight between ‘Fuji’ apple and ‘Gala’ apple (*p* < 0.05). The single fruit weight of almost 80% ‘Fuji’ apples fell within the range of 200–300 g and 80% ‘Gala’ apples showed single fruit weight within 100–200 g. The average single fruit weight of ‘Fuji’ apples (241.05 g) was 1.4 times as much as ‘Gala’ apples (169.48 g). Similar results have been found in previous studies (Du, 2023; Kuang et al., 2021; Zhang, 2018). For the same cultivar, there was no significant difference in single fruit weight between LP and BB regions. The obtained single fruit weight data of ‘Fuji’ and ‘Gala’ apple samples indicated that the cultivar had significantly effects on the apple fruit weight, which was in consistent with Argenta et al.'s finding (Argenta et al., 2022).

The fruit shape index is another important evaluation index (Du, 2023). Fruit shape index is calculated by measuring the ratio of vertical and horizontal diameter of the fruit, which can objectively reflect the shape, size and symmetry of the fruit, so as to evaluate the appearance quality of the fruit. In general, the fruit shape index of apples ranges from 0.8 to 0.9, resulting in a round or nearly round appearance. When the index is between 0.6 and 0.8, the fruit is oblate. Between 0.9 and 1.0, the fruit is either elliptical or conical. If the shape index exceeds 1.0, the fruit is considered elongated (Du, 2023). In the study, for ‘Fuji’ apples, the fruit shape index ranged from 0.77 to 1.03 and the average value was 0.875 (Fig. 1). Only 3 samples had a fruit shape index <0.8 and 1 sample had a fruit shape index higher than 1.0. The proportion of samples with fruit type index in the range of 0.8–0.9 and 0.9–1.0 was 76.3% and 21.5%, respectively. For ‘Gala’ apples, the fruit shape index ranged from 0.81 to 0.98 with the average value of 0.888 and 68.8% and 31.2% of ‘Gala’ apples were in the range of 0.8–0.9 and 0.9–1.0, respectively. The results showed that most of the ‘Fuji’ and ‘Gala’ apples were round or nearly round and the fruit type index of apples from LP origin was higher than that from BB origin.

Fruit firmness is a crucial parameter in assessing post–harvest quality of apples (Xu et al., 2021). The flesh firmness of apples depends on the strength of cell–to–cell adhesion, which is closely related to pectin and fiber content within the cell walls. The fruit firmness of ‘Fuji’ apples ranged from 5.54 to 9.89 kg/cm^2^ with the average value of 7.06 kg/cm^2^. For ‘Gala’ apples, the flesh firmness was from 5.16 to 10.63 kg/cm^2^ with the average value of 7.22 kg/cm^2^. In our previous study, the flesh firmness of ‘Fuji’ apples in China ranged from 4.69 to 10.58 kg/cm^2^ and the average flesh firmness was 6.93 kg/cm^2^ (Kuang et al., 2020). Du (2023) reported that the average flesh firmness of ‘Gala’ apples was 7.99 kg/cm^2^. The flesh firmness of ‘Gala’ apples from BB region (8.28 kg/cm^2^) was highest, followed by ‘Fuji’ apples from LP region (7.27 kg/cm^2^), ‘Gala’ apples from LP region (6.68 kg/cm^2^) and ‘Fuji’ apples from BB region (6.83 kg/cm^2^) were the lowest and there was no significant difference between ‘Gala’ apples from LP region and ‘Fuji’ apples from BB region.

### 3.2 Taste characteristics of different apple cultivars and origins

Although many fruits are graded using the external attributes, evaluated based on objective or subjective means, the internal quality characteristics, such as the most important internal qualities for apple's taste, sugar and sour, have strong influences on the consumer acceptance of fruits (Xu et al., 2021). As shown in Fig. 1, ‘Fuji’ apples showed a significantly higher TSS, SS and SV content than ‘Gala’ apples (*p* < 0.05). There was no significant difference between LP and BB regions in terms of TSS, SS, SV and TA in ‘Fuji’ apples. For ‘Gala’ apple, only TSS and SS showed no significant difference between two regions. The SV value of ‘Gala’ apples from LP origin was higher than BB origin and TA value of ‘Gala’ apples from LP origin was lower than BB origin.

Sugar–acid ratio is a critical indicator of fruit flavor quality, and there are substantial differences in sugar–acid ratio between different apple cultivars. A superior tasting apple typically has a sugar–acid ratio between 20 and 60. Fruits with ratios below 20 tend to be bland or tart, while those above 60 exhibit a strong sweet taste. In this research, 96% of the ‘Fuji’ apples and 97% of ‘Gala’ apples had a sugar to acid ratio in the 20–60 range. The TST and SVT of ‘Fuji’ apples were significantly higher than those of ‘Gala’ apples for the same region and the SST value didn't differ between cultivars. For same cultivar, the TST, SST and SVT of apples from LP region were significantly higher than those from BB region. The research of Zhang (2018) showed that TST value of ‘Fuji’ apples from BB and LP origins were 66.25 and 74.9, respectively, and the results of variance analysis between two origins was consistent with our research results.

**Fig. 2:**
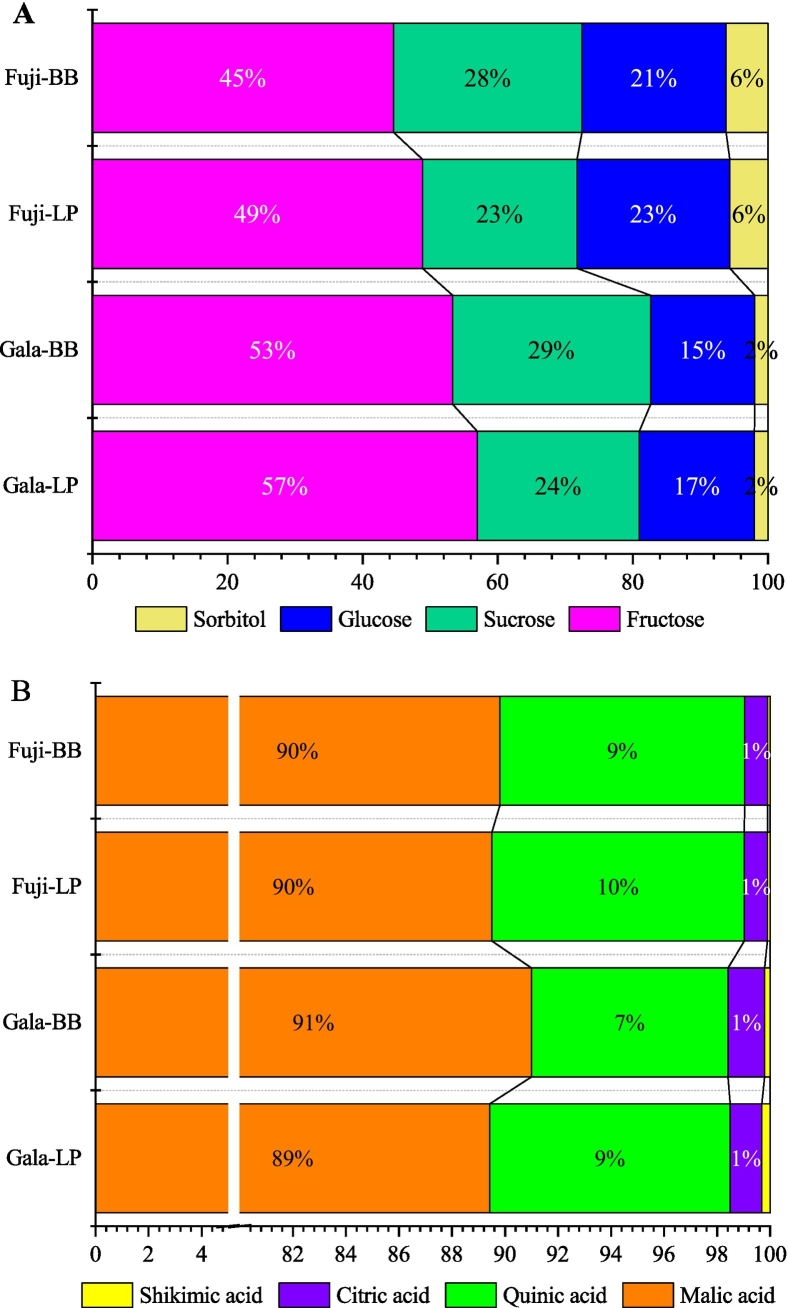
The proportion of each component in the total content. A: Soluble sugar; B: Organic acid.

In apples, soluble sugars primarily consist of sorbitol, glucose, fructose and sucrose. Fructose is the most abundant sugar in most apple varieties (Kim et al., 2023; Kuang et al., 2020; Zhang, 2022). Through the comparative analysis of 4 kinds of sugar components in apples, the fructose content was the highest, accounting for about 43.5–65.2% of the total sugar components, and the sorbitol content was the lowest, accounting for about 1.17–10.8% (Fig. 2A). ‘Fuji’ apples showed a relatively higher sorbitol, glucose, fructose and sucrose content than ‘Gala’ apples from the same region. The special sugar components varied greatly in different regions. There was no significant difference in sorbitol content between LP and BB regions. The glucose and fructose content of ‘Fuji’ and ‘Gala’ apples from LP was significantly higher than the apples from BB region. However, the sucrose content of ‘Fuji’ apples from LP region was the lower than that from BB region.

Organic acid is one of the most important components of fruit taste, and impacts on the overall organoleptic quality of apple fruit (Ma et al., 2018). In this study, the content of four acid components in apples was ranked as malic acid > quinic acid > citric acid > shikimic acid, and malic acid content accounted for about 90% of the total organic acid content (Fig. 2B), which was consistent with the results of previous studies on cultivated apple fruits (Ma et al., 2018; Yan et al., 2018; Zhang, 2022; Zhang et al., 2022). However, for some wild apple species, the citric acid content was higher than malic acid (Ma et al., 2018). Except shikimic acid, ‘Fuji’ apples showed a relatively higher malic acid, quinic acid and citric acid content than ‘Gala’ apples for the same region. There was no significant difference between the two regions regarding quinic acid, citric acid and shikimic acid content for ‘Fuji’ apple and quinic acid for ‘Gala’ apple. The malic acid content in two apple cultivars and citric acid content in ‘Gala’ apples from BB were significantly higher than the apples from LP region, while the opposite results appeared for shikimic acid of ‘Gala’ apples. As the major contributor to their acidity, malic acid directly influences an apple's taste, flavor, and preservation properties. Generally, a higher content of malic acid results in a better texture and more intense flavor in apples.

### 3.3 Nutrition characteristics of different apple cultivars and origins

Recently, the interest in fruit compositions has increased because of the awareness of their potential benefits for human health (Acquavia et al., 2021; Patocka et al., 2020). Apples are known for having significant amounts of vitamin C and phenolic compounds which have strong antioxidant activity and play a critical role in the prevention of various diseases such as cardiovascular diseases, neuropathies, and diabetes (Cantero et al., 2022; Kumari et al., 2023; Shahidi, 2020). It was confirmed that regular use of apples in a diet contributes in a significant way to the intake of phenolics (Joshipura et al., 2001). Especially for the class of flavonoids, apples turned out absolutely as one of the most important dietary sources (Wolfe et al., 2003).

Vitamin C contained in apples can improve the human immunity, prevent anemia, and act as antioxidant and anti–aging agents (Patocka et al., 2020). The average vitamin C content of ‘Fuji’ apples (37.68 mg/kg) was almost doubled than ‘Gala’ apples (19.74 mg/kg) (Table S1 and Fig. 1). In BB and LP origins, the vitamin C content of ‘Fuji’ apple was 1.6 times and 2.1 times than ‘Gala’ apple, respectively. The contents of vitamin C in ‘Fuji’ apples were not significantly different among the two regions. While for ‘Gala’ apples, the vitamin C content from BB region (24.18 mg/kg) was significantly higher than the apples from LP region (17.48 mg/kg). The vitamin C content of different apple cultivars has been reported by several studies. Kuang et al. (2020) evaluated 15 quality indicators of‘Fuji’ apples from 8 major producing provinces in China and the vitamin C content of ‘Fuji’ apples from different origins of China varied greatly, which ranged from 8.2 to 108.8 mg/kg. In Ningxia irrigation area of China, the vitamin C content of ‘Fuji’ and ‘Gala’ apples was 44.7 mg/kg and 66.0 mg/kg, respectively (Yang et al., 2022). The results showed that the cultivars and origins had great influences on the contents of vitamin C in apples.

The total phenolic content ranged from 164.98 to 539.83 mg GAE/kg FW in ‘Fuji’ apples with the average value of 322.66 mg GAE/kg FW. For ‘Gala’ apples, the total phenolic content was between 204.79 and 642.93 mg GAE/kg FW and the average value was 384.82 mg GAE/kg FW. There was no significant difference in the total phenolic content among ‘Gala’ apple from LP region, ‘Gala’ apple from BB region and ‘Fuji’ apple from LP region, which were significantly higher than ‘Fuji’ apple from BB region. The flavonoid content varied from 121.6 to 452.53 mg CE /kg FW in ‘Fuji’ apples and from 112.84 to 434.96 mg CE /kg FW in ‘Gala’ apples. The content of total flavonoid was the highest in ‘Fuji’ apple from LP region (285.37 mg CE /kg FW), followed by the ‘Gala’ apples from BB region (251.32 mg CE /kg FW) and LP region (249.33 mg CE /kg FW). The ‘Fuji’ apple from the BB region (208.78 mg CE /kg FW) had the lowest flavonoid concentration. 3.4. Principal component analysis.

**Fig. 3:**
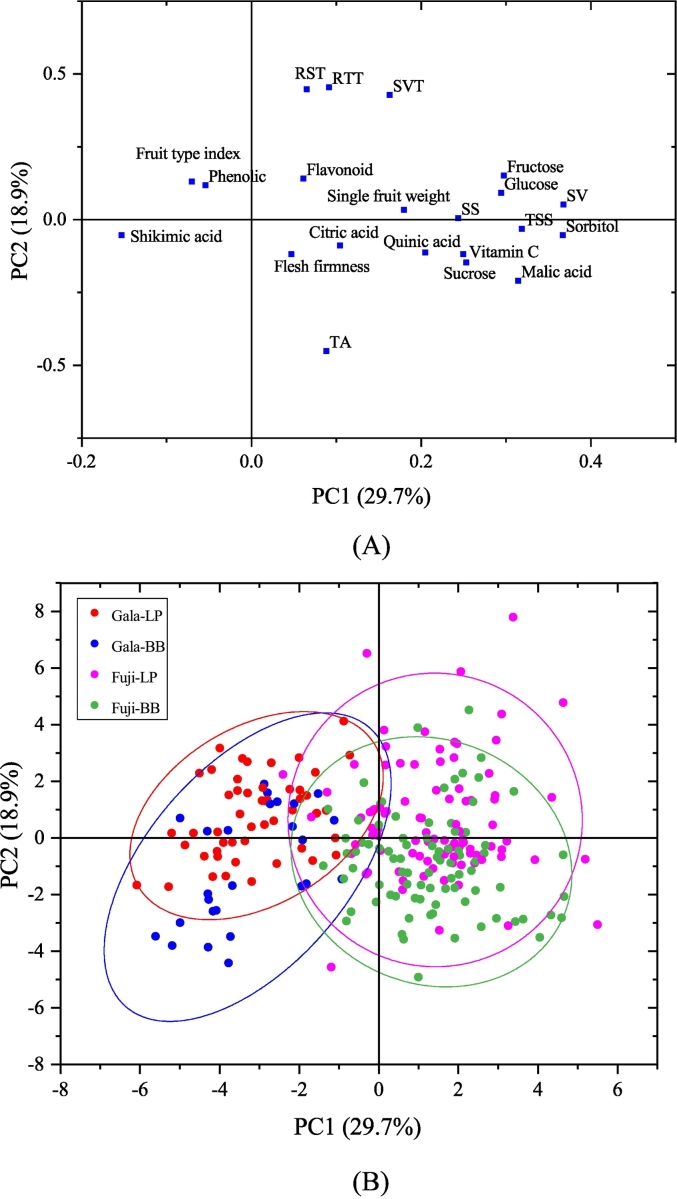
The PCA result of ‘Fuji’ and ‘Gala’ apples from Loess Plateau (LP) and Bohai Bay (BB) regions. A: Loading plot; B: Score plot.

Based on the 21 quality indicators in the apples, PCA was performed to construct several uncorrelated principal components (PCs) to reduce the data dimensionality. The cumulative variance contribution rate of the first seven PCs was 81.2%, which represented most of the information on all indexes. PC1 and PC2 accounted for 29.7% and 18.9% of the total variance, respectively. The PC1 represented the maximum variation of the data set. From Fig. 3A, except fruit type index, total phenolic and shikimic acid, the other indexes distributed in the positive region of PC1, indicating that they had the positive correlation with PC1. The main representative indicators for PC1 included single fruit weight, TSS, SS, SV, sorbitol, glucose, fructose, sucrose, malic acid, quinic acid, citric acid and Vc. To visualize the data trends, a score plot was obtained using the first two PCs. As shown in Fig. 3B, although some of the samples overlapped, the trend of the discrimination of samples based on their cultivar was evident. The ‘Gala’ samples were concentrated on the negative part of the PC1 coordinate, and the ‘Fuji’ samples were concentrated on the positive part, which showed that the content of representative indicators of PC1 in ‘Fuji’ samples was higher than that in ‘Gala’ samples.

### 3.4 3.5. Discriminant analysis

Apples of different cultivars or geographical origins could be easily mixed during harvesting and marketing. Therefore, effective and reliable technologies that could identify apple cultivar and geographical origin are demanded urgently by sellers and consumers (Li et al., 2018). In order to validate the characteristics of ‘Fuji’ and ‘Gala’ apples from different origins in terms of the physicochemical quality, linear and nonlinear discriminant analysis models for different cultivars and origins were established.

The linear discrimination functions for ‘Fuji’ and ‘Gala’ apples from different origins were established by stepped–linear discriminant analysis and the cross–validation was adopted to verify the accuracy of the models. According to the importance of each index to the model, the variables selected to participate in the modeling were malic acid (X_1_), SVT (X_2_), flesh firmness (X_3_), fruit type index (X_4_), fructose (X_5_), glucose (X_6_), phenolic (X_7_), flavonoid (X_8_), Single fruit weight (X_9_), SV (X_10_), shikimic acid (X_11_) and vitamin C (X_12_), respectively. Other variables did not participate in modeling due to their small contribution. Fisher's linear discriminant function were:

$$Y_{\left(\text{Gala}-\mathrm{LP}\right)}=-1.686\times {\;}_{1}+0.526\times {\;}_{2}+17.333\times {\;}_{3}+852.881\times {\;}_{4}+10.910\times {\;}_{5-}11.595\times {\;}_{6}+0.146\times {\;}_{7-}0.124\times {\;}_{8}+0.149\times {\;}_{9-}0.439\times {\;}_{10}+4816.274\times {\;}_{11}+1.300\times {\;}_{12}–508.783.$$

$$Y_{\left(\text{Gala}-\mathrm{BB}\right)}=-1.276\times {\;}_{1}+0.615\times {\;}_{2}+22.372\times {\;}_{3}+832.746\times {\;}_{4}+6.344\times {\;}_{5}–12.368\times {\;}_{6}+0.167\times {\;}_{7}–0.152\times {\;}_{8}+0.161\times {\;}_{9}–0.505\times {\;}_{10}+3783.811\times {\;}_{11}+1.385\times {\;}_{12}–508.327.$$

$$Y_{\left(\text{Fuji}-\mathrm{LP}\right)}=7.642\times {\;}_{1}+0.850\times {\;}_{2}+16.973\times {\;}_{3}+830.291\times {\;}_{4}+9.303\times {\;}_{5}–4.068\times {\;}_{6}+0.070\times {\;}_{7}–0.043\times {\;}_{8}+0.208\times {\;}_{9}–1.033\times {\;}_{10}+4010.811\times {\;}_{11}+2.547\times {\;}_{12}–533.318.$$

$$Y_{\left(\text{Fuji}-\mathrm{BB}\right)}=8.128\times {\;}_{1}+0.826\times {\;}_{2}+16.079\times {\;}_{3}+786.602\times {\;}_{4}+2.685\times {\;}_{5}–2.869\times {\;}_{6}+0.055\times {\;}_{7}–0.050\times {\;}_{8}+0.209\times {\;}_{9}+1.351\times {\;}_{10}+3776.522\times {\;}_{11}+2.241\times {\;}_{12}–482.912.$$

**Fig. 4:**
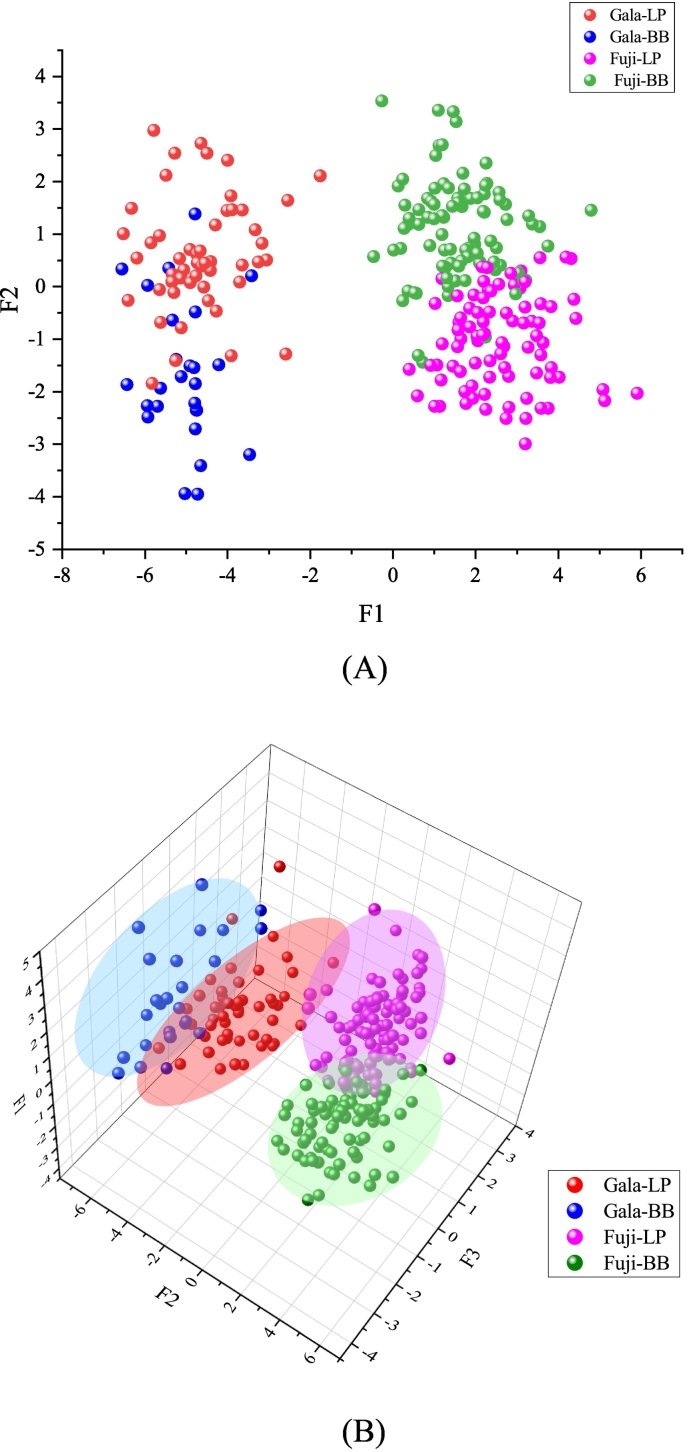
The LDA result of ‘Fuji’ and ‘Gala’ apples from Loess Plateau (LP) and Bohai Bay (BB) regions. A: Two–dimensional scatter plots; B: Three–dimensional scatter plots.

The separation of ‘Fuji’ and ‘Gala’ apples from Loess Plateau (LP) and Bohai Bay (BB) regions checked by plotting the function scores (Fig. 4). As could be seen from Fig. 4, the identification accuracy between cultivars was higher than that between origins. There was a clear distinction between ‘Fuji’ and ‘Gala’ apples. For the same cultivar, a few samples overlapped between BB and LP regions. The LDA classification and cross–validation results were summarized in Table 1. The total empirical grouped observations classification rate was 96.5%. The identification accuracy of ‘Fuji’ apple from BB and LP origins was 98.8% and 95.7%, respectively. The identification accuracy of ‘Gala’ apple from BB and LP origins was 96.2% and 94.1%, respectively. The cross–validated grouped observations classification rate was 92.9%. The classification rates of ‘Fuji’ apple from BB and LP origins was 95.3% and 91.3%, respectively. The classification rates of ‘Gala’ apple from BB and LP origins was 92.3% and 92.2%, respectively.

**Table 1:** Classification of ‘Fuji’ and ‘Gala’ apples from Loss Plateau and Bohai Bay regions by LDA.

		Gala–LP	Gala–BB	Fuji–LP	Fuji–BB	Rate of classification (%)
Original	Gala–LP	48	2	1	0	94.1
	Gala–BB	1	25	0	0	96.2
	Fuji–LP	0	0	88	4	95.7
	Fuji–BB	0	0	1	84	98.8
	Total					96.5
Cross–validated	Gala–LP	47	3	1	0	92.2
	Gala–BB	2	24	0	0	92.3
	Fuji–LP	0	0	84	8	91.3
	Fuji–BB	0	0	4	81	95.3
	Total					92.9

LDA: Linear discriminant analysis model; Gala–LP: ‘Gala’ apples grown in Loss Plateau region; Gala–BB: ‘Gala’ apples grown in Bohai Bay region; Fuji–LP: ‘Fuji’ apples grown in Loss Plateau region; Fuji–BB: ‘Fuji’ apples grown in Bohai Bay region.

**Fig. 5:**
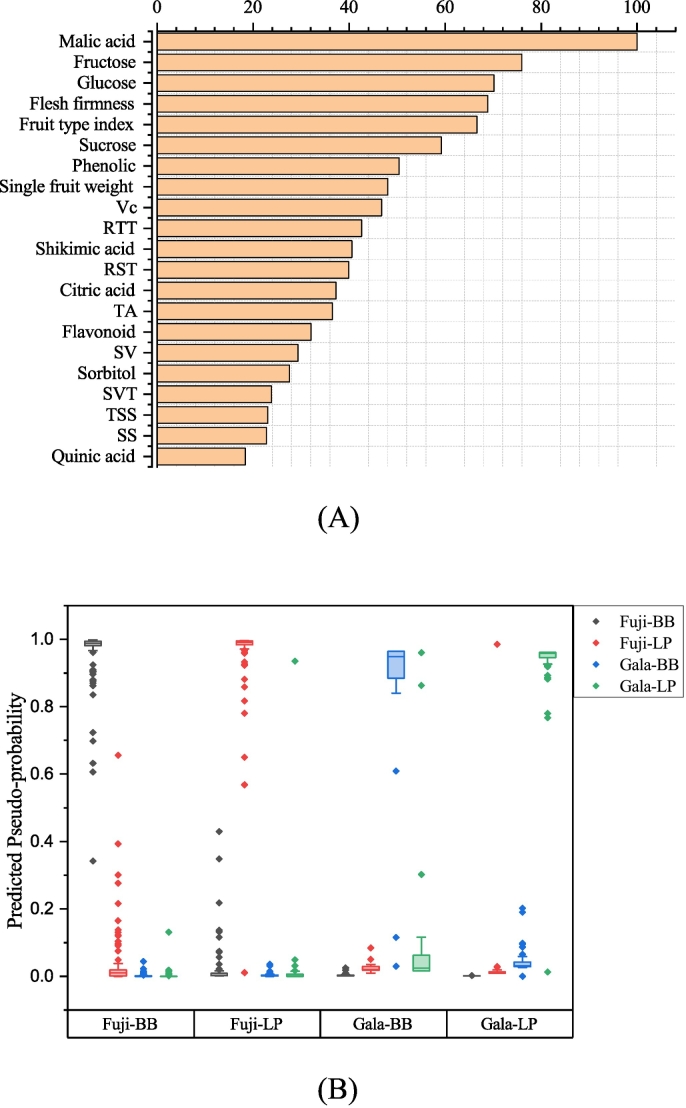
The MLP–NN result of ‘Fuji’ and ‘Gala’ apples from Loess Plateau (LP) and Bohai Bay (BB) regions. A: Relative importance of the indexes; B: Observation prediction plot.

**Fig. 6:**
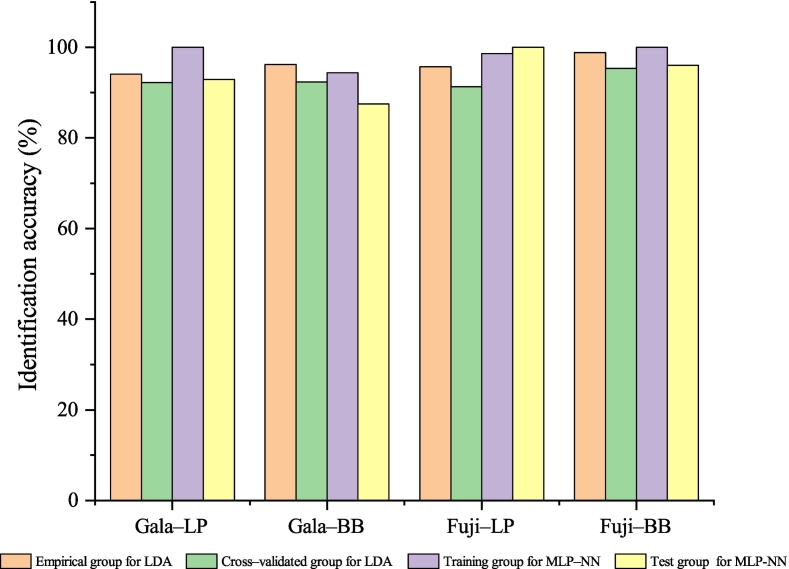
Comparison of discriminant results of LDA and MLP–NN models.

The nonlinear discrimination functions for ‘Fuji’ and ‘Gala’ apples from different origins were established by MLP–NN. The values of variable importance in projection for 21 quality indicators were calculated in the MLP–NN model (Fig. 5A). Malic acid, fructose, glucose, flesh firmness, fruit type index, sucrose, phenolic, single fruit weight, Vc, RTT, shikimic acid, and RST were at the top of the list, suggesting that these indicators have significant contributions to modeling. It could be seen from Fig. 5B, only 1 Fuji–BB sample, 1 Fuji–LP sample, 2 Gala–BB samples and 1 Gala–LP sample had the predicted pseudo–probability of <0.5, which indicated that the 5 samples were identified incorrectly. From Table 2 and Fig. 6, the average identification rate was 98.9% for the training set and 95.7% for the test set. For the training set, the identification accuracy of ‘Fuji’ apple from BB and LP origins was 100% and 98.6%, respectively. The identification accuracy of ‘Gala’ apple from BB and LP origins was 94.4% and 100%, respectively. For the test set, the classification rates of ‘Fuji’ apple from BB and LP origins was 96.0% and 100%, respectively. The classification rates of ‘Gala’ apple from BB and LP origins was 87.5% and 92.9%, respectively. Compared the results of the LDA and MLP–NN, the MLP–NN model generally obtained higher training and validation accuracy than that of the LDA model.

**Table 2:** Classification of ‘Fuji’ and ‘Gala’ apples from Loss Plateau and Bohai Bay regions by MLP–NN.

		Gala–LP	Gala–BB	Fuji–LP	Fuji–BB	Rate of classification (%)
Training (184)	Gala–LP	37	0	0	0	100
	Gala–BB	1	17	0	0	94.4
	Fuji–LP	1	0	68	0	98.6
	Fuji–BB	0	0	0	60	100
	Total					98.9
Test (70)	Gala–LP	13	0	1	0	92.9
	Gala–BB	1	7	0	0	87.5
	Fuji–LP	0	0	23	0	100
	Fuji–BB	0	0	1	24	96.0
	Total					95.7

MLP–NN: Multilayer perceptron neural network model; Gala–LP: The ‘Gala’ apples grown in Loss Plateau region; Gala–BB: The ‘Gala’ apples grown in Bohai Bay region; Fuji–LP: The ‘Fuji’ apples grown in Loss Plateau region; Fuji–BB: The ‘Fuji’ apples grown in Bohai Bay region.

## 4 Discussion

Due to its pleasant taste and high nutritional values, apples become one of the most popular fruits consumed worldwide. Fruit quality is one of the crucial factors in assessing the economic value of apples, encompassing both their appearance and inner attributes. Single fruit weight and fruit shape index are indicators of exterior quality. The intrinsic qualities mainly includes sugar content, acid content, vitamins, phenolics and flavonoids. Among the intrinsic qualities of fruits, sugars and acids are the primary flavor compounds. The composition and concentration of these elements significantly influence a fruit's sweetness, tartness, mouthfeel, and color (Xu et al., 2020). Apples with good appearance and internal quality usually have high commercial and economic value. Apples with good appearance quality can attract the eye of consumers and make them more willing to buy. Apples with good intrinsic quality can provide better taste and nutritional value to meet the needs of consumers.

There are many factors affecting the qualities of apple fruit, including the genetic characteristics of cultivars, ecological conditions and orchard management. Cultivars and ecological conditions play key roles in the formation of fruit quality. There are substantial differences in apple qualities between apple cultivars and between apple origins (Arnold & Gramza-Michalowska, 2023). In our study, 86% quality indicators were significantly different between ‘Fuji’ and ‘Gala’ apples. In LP origin, the value of single fruit weight, flesh firmness, Vc, TSS, SS, SV, TA, TST, SVT, flavonoids, quinic acid, malic acid, citric acid, sorbitol, glucose, fructose and sucrose of ‘Fuji’ apples was higher than ‘Gala’ apples. In BB origin, the value of single fruit weight, Vc, TSS, SS, SV, TST, SVT, quinic acid, malic acid, citric acid, sorbitol, glucose, fructose and sucrose of ‘Fuji’ apples was higher than ‘Gala’ apples and the values of flesh firmness, fruit shape index, total phenolic and flavonoid of Fuji apples were lower than ‘Gala’ apples.

Many scholars have studied some quality characteristics of ‘Fuji’ and ‘Gala’ apples. Zhang, Nock, et al. (2019) reported that the SS content and flesh firmness of ‘Fuji’ apples from the Cornell University orchards were higher than ‘Gala’ apples. Argenta et al. (2022) reported that single fruit weight, flesh firmness, soluble solid and titratable acidity content in ‘Fuji’ and ‘Gala’ apples grown in São Joaquim, Caçador, and Vacaria, Brazil. The results indicated that the single fruit weight and soluble sugar content of ‘Fuji’ apples were higher than those of ‘Gala’ apples in the 3 origins. The flesh firmness of ‘Fuji’ apples was higher than ‘Gala’ apples in São Joaquim, while the opposite results appeared in Caçador. The TA content of ‘Fuji’ apple was greater than that of ‘Gala’ in Caçador and Vacaria, while there was no significant difference in TA content between ‘Fuji’ and ‘Gala’ apples from Vacaria. Li et al. (2021) found that the contents of fructose, sucrose, SS, SV, malic acid and titratable acid in ‘Gala’ apples were higher than that in ‘Fuji’ apples and sorbitol, glucose, TSS, SST and SVT value in ‘Gala’ apple was lower than that in ‘Fuji’ apple. Mansoor et al. (2023) reported the total phenolic and flavonoid contents in 25 apple cultivars and the average content in ‘Fuji’ and ‘Gala’ apples was 64.79 and 80.98 mg GAE/100 g FW, respectively. The average flavonoid content in ‘Fuji’ and ‘Gala’ apples was 143 and 108 mg CE /100 g FW. Shafi et al. (2019) found that the total phenolic and flavonoid content in ‘Fuji’ apples were 174.8 mg GAE/kg and 0.108 mg QEA/g FW, respectively. Preti and Tarola (2021) reported that the total phenolic content in pulp of Royal Gala and ‘Fuji’ were 0.62 mg GAE/g FW and 0.22 mg GAE/g FW. Based on the above research results, ‘Fuji’ apples were larger, sweeter or have a higher sweet–sour ratio than ‘Gala’ apples in most origins. Our results confirmed this.

Apples from different regions exhibit distinct quality characteristics. Between LP and BB origins, there were significantly differences for ‘Gala’ apples regarding flesh firmness, fruit shape index, Vc, SV, TA, TST, SST, SVT, malic acid, citric acid, shikimic acid, glucose, fructose or sucrose for Gala apples. For Fuji apples, there were significantly differences in phenolic, TST, SST, SVT, flavonoid, malic acid, fructose and sucrose. Zhang (2022) compared the qualities of ‘Fuji’ apples grown in BB and LP origins, the results indicated that TSS content of apples from the LP origin was significantly higher than that of the BB origin, while the TST was significantly lower than that of BB origin. There were no significant differences in single fruit weight, fruit shape index, flesh firmness and TA content between BB and LP origin. Another study showed that the fructose content of ‘Fuji’ apples from the LP origin was higher than BB origin, while the opposite results appeared for sucrose. There was no significant differences between BB and LP origins for sorbitol, glucose, malic acid, quinate acid and shikimic acid (Zhang, 2022). The results showed that the origin environment had great influence on apple qualities.

Guo (2006) studied the correlations between ecological environment and ‘Fuji’ and ‘Gala’ apple qualities, finding that the annual mean temperature had positive correlations with single weight and fruit shape index, and negative correlations with fruit firmness, soluble solids, total acid, vitamin C and anthocyanin for the ‘Gala’ apple. And for the ‘Fuji’ apple, the annual mean temperature had positive correlations with single weight, and negative correlations with fruit firmness, fruit shape index, soluble solids, total acid, vitamin C and anthocyanin. To annual year rainfall, and for the ‘Gala’ apple, there was a positive correlation with single weight, and negative correlations with fruit firmness, total acid, vitamin C, fruit shape index and anthocyanin. For the ‘Fuji’ apple, annual year rainfall showed a positive correlation with single weight and negative correlations with fruit firmness, soluble solids, total acid, vitamin C, and fruit shape index. For the average relative humidity from June to August, for the ‘Gala’ apple, there was a positive correlation with single weight and anthocyanin. There were negative correlations with fruit firmness, soluble solids, total acid, vitamin C and fruit shape index. And for the ‘Fuji’ apple, there was a positive correlation with single weight and anthocyanin. There were negative correlations with fruit firmness, soluble solids, total acid, vitamin C and fruit shape index. To the elevation, for the ‘Gala’ apple, there were positive correlations with vitamin C, fruit firmness, soluble solids, total acid and anthocyanin. There were negative correlations with single weight, fruit shape index. And for the ‘Fuji’ apple, there were positive correlations with soluble solids, fruit firmness, total acid, vitamin C and anthocyanin, which got to notable levels. There were negative correlations with single weight, fruit shape index, which got to notable levels. A comprehensive analysis revealed that the impact of meteorological factors on fruit quality was quite complex. Different geographical locations, altitudes, varieties, and growth seasons all experience varying influences from these atmospheric elements.

Soils, as the foundation for tree growth and development, directly affect the apple yield and fruit qualities. Elements such as nitrogen, potassium, and calcium in soils can influence apple qualities. Han et al. (2018) conducted a multivariate analysis of soil nutrients and fruit quality in 31 typical orchards in the southern Taihang Mountains of China, indicating that soil organic matter was positively correlated with fruit solid–acid ratio, soil available copper was negatively correlated with single fruit weight and fruit shape index, but positively correlated with fruit firmness and vitamin C, soil available manganese was positively correlated with vitamin C and fruit firmness, soil available zinc was positively correlated with single fruit weight and soluble solids. Zhang (2018) investigated the relationship between ‘Fuji’ apple quality attributes and soil nutrient factors as well as meteorological factors. The findings revealed that meteorological factors had a more significant impact on fruit quality (63.87%) compared to soil nutrients (36.13%). Optimal growing regions were identified as a priority for enhancing apple quality.

These influencing factors are generally similar within a certain region. Therefore, these difference indicators carry certain information about the origin, and they can be used to trace the source of the apples. Tan et al. (2023) determined 24 quality components of ‘Fuji’ apples from three different provinces and established an origin discriminant model by combining single–factor variance analysis, principal component analysis, partial least squares discrimination, and linear discrimination analysis. The accuracy of the model's discrimination and verification exceeded 88%. Zhang (2022) established a tracing model of ‘Fuji’ apple from LP and BB origins based on the contents of sorbitol, glucose, fructose and sucrose. The correct classification rate of samples was 79.2%, and the cross–verification rate was 75.3%. There are a few reports on the origin discrimination of ‘Fuji’ apple, but there is no report on the origin discrimination analysis of ‘Gala’ apple. In this study, linear and nonlinear identification models were established for ‘Fuji’ and ‘Gala’ apples from two dominant origins, which could be used to identify cultivars and origins, simultaneously.

## 5 Conclusion

The aim of this study was to explore the cultivar and region quality characteristics of ‘Fuji’ and ‘Gala’ apples from two dominant origins in China and the viability of tracing the cultivar and geographical origin. Twenty–one quality properties in apple fruits were analyzed and LDA and MLP–NN models were constructed for apple cultivar and origin authentication. The results indicated that 85.7% quality indicators were significantly different between ‘Fuji’ and ‘Gala’ apples from the same origin. Between LP and BB origins, there were significantly differences regarding 66.7% indicators for ‘Gala’ apples and 38.1% indicators for ‘Fuji’ apples. The total empirical grouped observations classification rate was 96.5% and the cross–validated grouped observations classification rate was 92.9% for LDA model. The MLP–NN model performed well in discriminating different source cultivars and regions, with an average identification rate of 98.9% for the training set and 95.7% for the test set. The research has certain theoretical significance and practical value for the production, consumption, breed selection, and quality evaluation of apples.

## CRediT authorship contribution statement

**Lixue Kuang:** Software, Methodology, Formal analysis. **Zhiqiang Wang:** Writing – review & editing, Conceptualization. **Yang Cheng:** Software, Methodology. **Haifei Li:** Writing – review & editing, Data curation. **Jing Li:** Writing – review & editing, Formal analysis. **Youming Shen:** Writing – review & editing, Data curation. **Jianyi Zhang:** Writing – review & editing, Conceptualization. **Guofeng Xu:** Funding acquisition.

## Declaration of competing interest

The authors declare that they have no known competing financial interests or personal relationships that could have appeared to influence the work reported in this paper.

## Data Availability

No data was used for the research described in the article.

## Appendix A Supplementary data

Supplementary material Image 1
