# Supplementary material for: Cultivar and origin authentication of ‘Fuji’ and ‘gala’ apples from two dominant origins of China based on quality attributes
Source: Food Chem X. 2024 Jul 14;23:101643. doi: 10.1016/j.fochx.2024.101643 (PMC11637219; doi:10.1016/j.fochx.2024.101643)
Supplement: Supplementary file 1 — Supplementary material [file mmc1.docx]

Table S1 Quality comparison of ‘Fuji’ and ‘Gala’ apples from Loss Plateau and Bohai Bay regions.

| Index | Gala–LP | Gala–BB | Fuji–LP | Fuji–BB | Average value of Gala apple | Average value of Fuji apple |
| --- | --- | --- | --- | --- | --- | --- |
| Single fruit weight (g) | 174.00b | 161.22b | 239.12a | 243.13a | 169.48 | 241.05 |
| Fruit type index | 0.895a | 0.874b | 0.896a | 0.852c | 0.889 | 0.875 |
| Flesh firmness (kg/cm^2^) | 6.68c | 8.28a | 7.27b | 6.83c | 7.21 | 7.06 |
| TSS (%) | 12.68b | 12.82b | 14.81a | 14.57a | 12.73 | 14.69 |
| SS (%) | 10.91b | 10.56b | 11.99a | 11.72a | 10.79 | 11.86 |
| SV | 11.57b | 10.46c | 14.77a | 14.38a | 11.19 | 14.58 |
| TA (%) | 0.267b | 0.315a | 0.292a | 0.314a | 0.283 | 0.302 |
| RTT | 48.59b | 43.24c | 52.56a | 47.72b | 46.78 | 50.23 |
| RST | 41.67a | 35.56b | 42.7a | 38.23b | 39.6 | 40.5 |
| SVT | 44.61b | 36.01c | 52.52a | 47.17b | 41.7 | 50.0 |
| Sorbitol (%) | 0.171b | 0.152b | 0.662a | 0.724a | 0.164 | 0.692 |
| Glucose (%) | 1.45b | 1.2c | 2.64a | 2.48a | 1.363 | 2.566 |
| Fructose (%) | 4.83c | 4.16d | 5.71a | 5.2b | 4.603 | 5.463 |
| Sucrose (%) | 2.04c | 2.28c | 2.67b | 3.25a | 2.119 | 2.952 |
| Quinic acid (mg/g) | 0.187b | 0.175b | 0.447a | 0.462a | 0.183 | 0.455 |
| Malic acid (mg/g) | 1.84d | 2.15c | 4.21b | 4.5a | 1.94 | 4.35 |
| Shikimic acid (mg/g) | 0.00649a | 0.0049b | 0.00495b | 0.00461b | 0.00596 | 0.00479 |
| Citric acid (mg/g) | 0.0245c | 0.0326b | 0.0411a | 0.0438a | 0.0272 | 0.0424 |
| Vitamin C (mg/kg) | 17.48c | 24.18b | 37.68a | 38.31a | 19.74 | 37.98 |
| Phenolic (mg/kg) | 381.64a | 391.04a | 366.72a | 274.97b | 384.82 | 322.66 |
| Flavonoid (mg/kg) | 249.33b | 251.32b | 285.37a | 208.78c | 250 | 248.6 |

Gala–LP: The ‘Gala’ apples grown in Loss Plateau region; Gala–BB: The ‘Gala’ apples grown in Bohai Bay region; Fuji–LP: The ‘Fuji’ apples grown in Loss Plateau region; Fuji–BB: The ‘Fuji’ apples grown in Bohai Bay region; TSS: Total soluble solid; SS: Soluble sugar; SV: Sweetness value; TA: Titratable acidity; RTT: The ratio of total soluble solid to titratable acidity; RST: The ratios of soluble sugar to titratable acidity; SVT: The ratios of sweetness value to titratable acidity.

1. Physicochemical analyses

1.1 Titratable acidity

Weighed 25.0 g homogenized sample into a 250 mL conical bottle, added about 50 mL 80℃ no carbon dioxide water, after mixed well, boiled in boiling water bath for 30 min. Cooled the sample to room temperature and filled with no carbon dioxide water to 250 mL, then filtered with filter paper. The filtered liquid (25 ml) was titrated with 0.1 mol∙L^-1^ sodium hydroxide solution to the end point by the Automatic potentiometric titrator (904 Titrino, Metrohm, Switzerland).

1.2. Vitamin C

The Determination of vitamin C was conducted with the 2, 6–dichlor–oindophenol (2, 6–D) solution titration method (National Health Commission of the People’s Republic of China, 2016). The whole process was carried out under dark conditions. First of all, the titer of 2, 6-D was calculated. 1 mL of ascorbic acid solution (1 mg∙mL^-1^) and 10 mL metaphosphate solution are titrated by 2, 6–D solution until it turns pink, with the color holds for 15 s. The blank test was conducted with the metaphosphoric acid solution. And the titer of 2, 6–D solution was calculated by the following formula:

$$T=\frac{c\times V}{V_{1}-V_{0}}$$

T is the solution (mg∙mL^-1^) titer of 2, 6-dichloroindophenol; c is the solution concentration (mg∙mL^-1^) of ascorbic acid; V is the volume of ascorbic acid solution (mL); V_1_ is the 2, 6–D solution consumption of ascorbic acid solution titration (mL), while V_0_ is the consumption of blank test (mL).

The sample (100 g) was milled with 100 g metaphosphoric acid solution (20 g∙L^-1^), and 20 g of that was transferred to a 100 mL volumetric flask with metaphosphoric acid solution filling to the scale. 10 mL solution was titrated with 2, 6-D solution until the solution turns pink and holds for 15 s. The blank test was conducted with the metaphosphoric acid solution. Vitamin C concentration is calculated by the formula attached below:

$$X=\frac{(V-V_{0})\times T\times A}{m}\times100$$

X is the vitamin C concentration of sample (mg/100 g); V is the 2, 6–D solution consumption of sample test (mL), while V_0_ is the consumption of blank test (mL); T is the titer of 2, 6-D solution (mg∙mL^-1^); A is the dilution multiples; m is the sample weight (g).

1.3. Soluble sugar

The determination of soluble sugar (SS) was conducted with the 3, 5–dinitrosalicylic acid colorimetry method (Ministry of Agriculture, People’s Republic of China, 2015).

First of all, the 3, 5-dinitrosalicylic acid reagent was configured. Weighed 6.3 g 3, 5-dinitrosalicylic acid and 262 mL 2 mol∙L^-1^ sodium hydroxide solution into 500 mL hot water containing 185 g potassium sodium tartrate, then added 5 g phenol and 5 g sodium sulfite, stirred to dissolve, and filled with water to 1000 mL, stored in brown bottle for later use.

Weighed 10.00 g homogenized sample and transferred it to a 250 mL volumetric flask using water. After added 3 mL potassium ferrocyanide solution (0.106 g∙mL^-1^) and 3 mL zinc acetate solution (0.219 g∙mL^-1^), filled to the scale. Transferred 5 mL of the sample solution into a 100 mL volumetric flask and added 1 mL hydrochloric acid solution (6 mol∙L^-1^). The sample was then placed in a constant temperature water bath (HWS28, Shanghai Yiheng Instruments Co., Ltd., China) set to 80°C and extracted for 10 min. Then cooled and added 3 drops of methyl red indicator. The sample was neutralized to a light orange color using 6 mol∙L^-1^ sodium hydroxide solution and diluted to 100 mL with water, mixed thoroughly. After that, withdrew 1.0 mL of the sample solution to a 10 mL stoppered graduated cylinder, added 1.0 mL water and 4.00 mL 3,5-dinitrosalicylic acid reagent. After heated in a boiling water bath for 5 min, cooled it to room temperature and filled to the scale. Measure the absorbance value at 540 nanometers using a spectrophotometer. The content of soluble sugar in the sample was obtained according to the standard curve of glucose.

1.4. Soluble sugar components, organic acids, total phenolic and total flavonoid

Sample extraction: Weighed 5 g of sample was ground in 25 mL of 80% methanol and then extracted by ultrasound at room temperature (25°C) for 20 min. After centrifugation at 9 000 r∙min^–1^ for 5 min, the supernatant was poured into 50.0 mL brown volumetric bottle and filled to the scale with 80% methanol. Ten milliliter of solution were evaporated under vacuum at 40℃. The solution was nearly dried, and 5ml of water was added. The sample extraction liquid was poured into the activated solid phase extraction column. The filtrate was collected and then filled with water to 25 mL and passed through a 0.22 μm cellulose filter membrane for the determination of soluble sugar components and organic acids. Then the solid phase extraction column was washed with 5 mL methanol for two times, and the filtrate was collected for the determination of total phenolic and flavonoid.

The determination of soluble sugar components: The soluble sugar components (sorbitol, glucose, fructose, and sucrose) were detected by an Ion chromatograph (ICS–5000, Dionex, USA) with a conductivity detector, an anion exchange analytical column (Dionex CarboPacTM PA10, 4 mm × 250 mm, Thermo Fisher Scientific, USA), and a guard column (IonPac AG23, 4 mm × 50 mm, Dionex, USA).. The operating conditions were: mobile phase, 0.2 mol∙L^–1^ NaOH solution; flow rate, 1.0 mL∙min^–1^; column temperature, 30°C; and injection volume, 10 μL. Data processing and instrument control were carried out using Chromeleon Software.

The determination of organic acids: Four organic acid compounds, including quinic acid, malic acid, Citric acid and shikimic acid were detected by HPLC (LC–10A, Shimadzu, Japan), with SPD–10A UV–VIS detector, and a C18 column (Ultimate Loss Plateau–C18, 4.6 mm × 300 mm, 5 μm, Ultimate, China). The eluate was measured by UV absorption at 210 nm. The operating conditions were programmed as follows: mobile phase, 0.01 mol∙L^-1^ KH_2_PO_4_ solution; flow rate, 0.5 mL min^–1^; column temperature, 40℃; and injection volume, 10 μL.

The determination of total phenolic: First of all, sodium tungstate–sodium molybdate mixed solution was configured. Weighed 50.0 g sodium tungstate and 12.5 g sodium molybdate to a 1000 mL reflux bottle, added 350 ml water to dissolve. After added 25 mL phosphoric acid and 50 mL hydrochloric acid, heated the reflux for 2h on low fire, then added 75 g lithium sulfate, 25 mL distilled water, a few drops of bromine water, and continued boiling for 15min (until bromine water was completely volatilized). After cooling, transferred to 500 mL volumetric bottle and filled to the scale. Finally, the solution was store in brown bottle. Absorbed 1.0 mL sample extract solution to a 10 mL scale test tube, added 5.0 mL water, 1.0 mL sodium tungstate–sodium molybdate mixed solution and 3.0 mL sodium carbonate solution (75 g∙L^-1^), respectively. After two hours of storage, the absorbance of sample solution was determined at 765nm wavelength. The results were expressed as milligrams GA equivalent (GAE) per kilogram.

The determination of total flavonoid: Absorbed 1.0 mL sample extraction solutionto a 10 mL scale test tube, added 5.0 mL water, 0.2 mL sodium nitrite solution (50 g∙L^-1^), 0.2 mL aluminum chloride solution (100 g∙L^-1^), and 2 mL sodium hydroxide solution (1.0 mol∙L^-1^), and filled to 10 mL. The absorbance of sample solution is determined at a wavelength of 500 nm. The results were expressed in terms of the catechin equivalent CE (mg∙kg^-1^).
